# Supplementary material for: Clinical Features and Outcome of Multidrug-Resistant Osteoarticular Tuberculosis: A 12-Year Case Series from France
Source: Microorganisms. 2022 Jun 14;10(6):1215. doi: 10.3390/microorganisms10061215 (PMC9229793; doi:10.3390/microorganisms10061215)
Supplement: Supplementary file 1 [file microorganisms-10-01215-s001.zip › microorganisms-1766317-supplementary.pdf]

Table S1. Outcomes definitions for RR-TB/MDR-TB/XDR-TB patients treated with second-line treatment used in the study, according to the WHO guidelines [8]

**Outcome**	**Definition**
Cured	Treatment completed as recommended by the national policy without evidence of failure AND three or more consecutive cultures taken at least 30 days apart are negative after the intensive phase[1]
Treatment completed	Treatment completed as recommended by the national policy without evidence of failure BUT no record that three or more consecutive cultures taken at least 30 days apart were negative after the intensive phase[1]
Treatment success	The sum of *cured* and *treatment completed*
Treatment failed	Treatment terminated or need for permanent regimen change of at least 2 anti-TB drugs because of:<br>- lack of conversion[2] by the end of the intensive phase[1], *or*<br>- bacteriological reversion[2] in the continuation phase after conversion[2] to negative, *or*<br>- evidence of additional acquired resistance to fluoroquinolones or second-line injectable drugs, *or*<br>- adverse drug reactions (ADRs)[3].
Died	A patient who dies for any reason during the course of treatment.
Lost to follow-up	A patient whose treatment was interrupted for 2 consecutive months or more.
Not evaluated	A patient for whom no treatment outcome is assigned. (This includes cases “transferred out” to another treatment unit and whose treatment outcome is unknown).

[1] For *Treatment failed*, lack of conversion by the end of the intensive phase implies that the patient does not convert within the maximum duration of the intensive phase as applied by the programme. If no maximum duration is defined, an 8-month cut-off is proposed. For regimens without a clear distinction between intensive and continuation phases, a cut-off 8 months after the start of treatment is suggested to determine when the criteria for *Cured*, *Treatment completed* and *Treatment failed* start to apply.

[2] The terms “conversion” and “reversion” of culture as used here are defined as follows:

Conversion (to negative): culture is considered to have converted to negative when two consecutive cultures, taken at least 30 days apart, are found to be negative. In such a case, the specimen collection date of the first negative culture is used as the date of conversion.

Reversion (to positive): culture is considered to have reverted to positive when, after an initial conversion, two consecutive cultures, taken at least 30 days apart, are found to be positive. For the purpose of defining *Treatment failed*, reversion is considered only when it occurs in the continuation phase.

[3] Criteria of failure were not retained in our study because of its low relevance [10]
